# Endovascular Occlusion of Giant Posttraumatic Pseudo-Aneurysm of Superior Mesenteric Artery Connected to Mesenteric Arteriovenous Fistula

**DOI:** 10.17691/stm2020.12.4.07

**Published:** 2020-08-27

**Authors:** K.R. Dzhagraev, R.Sh. Muslimov, A.B. Klimov, V.E. Ryabukhin, T.E. Kim, I.E. Selina, V.P. Kiryushchenkov, V.A. Moskalenko, L.S. Kokov

**Affiliations:** Leading Researcher, Department of Emergency Surgery, Endoscopy and Resuscitation; N.V. Sklifosovsky Research Institute of Emergency Care, 3 Bolshaya Sukharevskaya Square, Moscow, 129090, Russia; Associate Professor, Department of Emergency and General Surgery; Russian Medical Academy of Continuous Professional Education, 2/1, Bldg 1, Barrikadnaya St., Moscow, 125993, Russia;; Leading Researcher, Department of Radiation Diagnostics; N.V. Sklifosovsky Research Institute of Emergency Care, 3 Bolshaya Sukharevskaya Square, Moscow, 129090, Russia;; Physician, Department of X-ray Surgery Diagnosis and Therapy; N.V. Sklifosovsky Research Institute of Emergency Care, 3 Bolshaya Sukharevskaya Square, Moscow, 129090, Russia;; Physician, Department of X-ray Surgery Diagnosis and Therapy; N.V. Sklifosovsky Research Institute of Emergency Care, 3 Bolshaya Sukharevskaya Square, Moscow, 129090, Russia;; Physician, 2^nd^ Surgery Department; N.V. Sklifosovsky Research Institute of Emergency Care, 3 Bolshaya Sukharevskaya Square, Moscow, 129090, Russia;; Leading Researcher, Department of Radiation Diagnostics; N.V. Sklifosovsky Research Institute of Emergency Care, 3 Bolshaya Sukharevskaya Square, Moscow, 129090, Russia;; Physician, Department of X-ray Surgery Diagnosis and Therapy; N.V. Sklifosovsky Research Institute of Emergency Care, 3 Bolshaya Sukharevskaya Square, Moscow, 129090, Russia;; Physician, Department of X-ray Surgery Diagnosis and Therapy; N.V. Sklifosovsky Research Institute of Emergency Care, 3 Bolshaya Sukharevskaya Square, Moscow, 129090, Russia;; Professor, Academician of the Russian Academy of Sciences, Head of the Department of Radiation Diagnostics; N.V. Sklifosovsky Research Institute of Emergency Care, 3 Bolshaya Sukharevskaya Square, Moscow, 129090, Russia; Head of the Radiodiagnosis Department, Institute of Professional Education I.M. Sechenov First Moscow State Medical University (Sechenov University), 8/2 Trubetskaya St., Moscow, 119991, Russia

**Keywords:** giant pseudo-aneurysm of superior mesenteric artery, traumatic mesenteric arteriovenous fistula, Amplatzer Vascular Plug II occluder, endovascular occlusion of mesenteric arteriovenous fistula.

## Abstract

**The aim of the study** was to show the capabilities of endovascular occlusion of giant posttraumatic pseudo-aneurysm of superior mesenteric artery (SMA) connected to a mesenteric arteriovenous fistula (AVF) under the conditions of portal hypertension and life-threatening esophageal variceal bleeding.

**Materials and Methods.:**

A 27-old male patient underwent endovascular occlusion; the patient being hospitalized with a clinical picture of gastrointestinal bleeding. The examinations: ultrasound, esophagogastroduodenoscopy, multispiral computed tomography with angiography — revealed the source of bleeding to be esophageal varices against the background of portal hypertension caused by massive arteriovenous shunt, its source being AVF with an aneurysmal component (32×35 mm in size) between SMA and superior mesenteric vein (SMV) dilated up to 50 mm in diameter. Patient’s past medical history recorded that 4.5 years ago the patient had undergone the resection of a small intestine area due to a penetrating stab wound in the abdominal cavity. Taking into consideration an extremely high operative intervention risk due to the condition severity related to blood loss, portal hypertension, and ascites, it was decided to embolize AVF with a vascular occluder — Amplatzer Vascular Plug II (USA), 14×10 mm in size.

**Results.:**

A unique endovascular intervention — transcatheter occlusion of pseudo-aneurysm and AVF separation — was performed in life-threatening esophageal variceal bleeding under the condition of a giant post-traumatic aneurysm of SMA and mesenteric AVF. Due to an extremely large-sized SMV and an arterial pseudo-aneurysm, first ever we used the technique applied for transcatheter occlusion of a cardiac septum defect.

Occluder implantation enabled to completely close the communication of aneurysmatic AVF with SMV, and occlude the aneurysm cavity. During an immediate postoperative period portal hypertension was arrested. No recurrent bleedings occurred within 4 postoperative months.

## Introduction

Traumatic injuries of major abdominal vessels result in life-threatening complications, which are difficult to eliminate completely and safely using an open surgery or laparoscopic operations. One of delayed sequelae of traumatic vessel injuries is an arteriovenous fistula (AVF) [1]. In most cases AVFs are detected in patients many years after an injury. Mesenteric AVF is a rare complication after surgeries or wounds. According to Brucher et al. [2], just about 200 observations of AVF between abdominal vessels have been described in literature. Mesenteric AVFs, as a rule, are accompanied by the portal system arterialization, portal hypertension development, esophageal and gastric varicose veins, with further complications: variceal bleedings [3].

Currently, there are no standard techniques to separate AVF, but at the same time increasingly frequently there have been reported on endovascular technologies as an alternative option of AVF occlusion, which enable to minimize infection and bleeding risks in open surgeries. Due to the variability of anatomy and size of intervascular fistulas, aneurysms of mesenteric vessels, the presence of portal hypertension and its complications, the therapy of such patients requires extraordinary, and sometimes unique solutions on using nonstandard approaches and devices — endovascular occluders.

**The aim of the study** was to show the capabilities of endovascular occlusion of giant posttraumatic pseudoaneurysm of superior mesenteric artery and a mesenteric arteriovenous fistula under the conditions of portal hypertension and a life-threatening esophageal variceal bleeding.

## Materials and Methods

We are going to represent a successful endovascular occlusion with an example of the therapy of a patient with a traumatic giant pseudo-aneurysm of superior mesenteric artery (SMA) and AVF between SMA and the vein complicated by secondary portal hypertension and esophageal and gastric variceal bleeding. The patient signed an informed consent to participate in the investigation.

*A 27-year-old male patient S. was admitted to N.V. Sklifosovsky Research Institute of Emergency Care with a suspected upper gastrointestinal bleeding, and complaints of abdominal pain of unclear localization.*

*On admission: the patient was in critical condition, undernourished. He was atonic, adynamic, had pale skin. AP was 85/60 mm Hg, pulse: 115 bpm, respiratory rate: 22. There were a medial postoperative scar and varicose subcutaneous veins on the anterior abdominal wall. Above the postoperative scar: systolic-diastolic thrill was palpable, and systolic-diastolic murmur was auscultated. Blood test showed: Hb — 74 g/L, posthemorrhagic anemia signs.*

*Past medical history: 4.5 years ago the patient underwent laparotomy and the resection of a small intestine area after a penetrating stab wound in the abdominal cavity. Three months prior to the hospitalization the patient had suffered from abdominal pains of unclear localization, fatigue, and dizziness. Two days before the admission he had had bloody vomiting and loose black stool, the patient feeling unwell.*

*Esophagogastroduodenoscopy (EGDS) showed active oozing hemorrhage in the esophago-gastric junction area, along the anterior wall, the mucosa was visually unchanged, the probable source of bleeding being esophageal varices. Blackmore tube was inserted.*

*Abdominal ultrasound revealed aneurysmal dilatation, which was regarded as an abdominal aortic aneurysm, 32×35 mm in size, at the level of supra- and intra-renal aortal area.*

**Figure 1:**
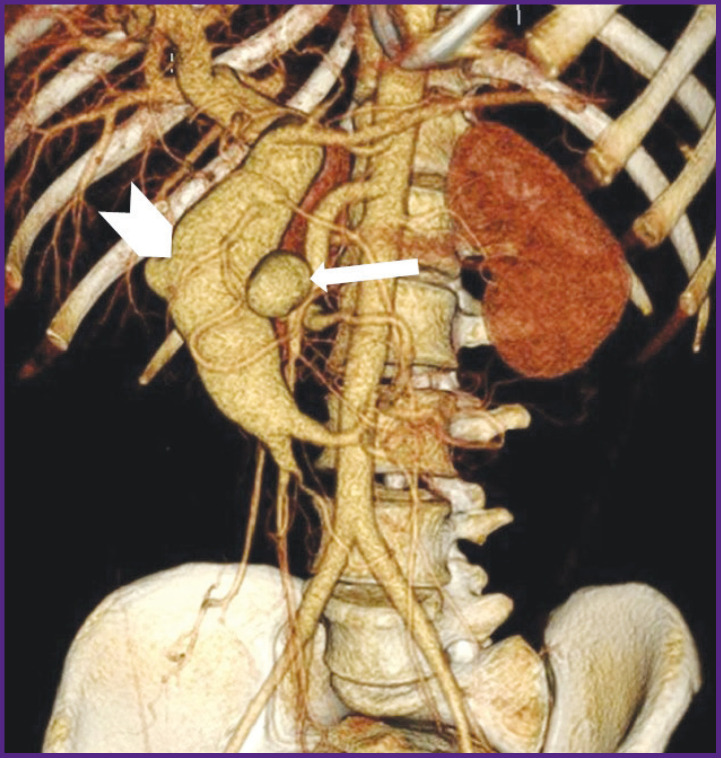
Patient S., MSCT-angiogram In proximal third of the superior mesenteric artery there can be seen a pseudo-aneurysm (*narrow arrow*) communicating with the superior mesenteric vein; the superior mesenteric vein lumen is enlarged up to 5 cm (*wide arrow*)

*According to abdominal multispiral computed tomography (MSCT) with contrast enhancement (Figure 1), in the abdominal cavity and true pelvis, there was free fluid (ascites), about 2000 ml in volume. The liver was not enlarged. In proximal SMA third, there was an ovoid-shaped pseudo-aneurysm, 32×35 mm in size, widely connected with the artery lumen due to the anterolateral wall defect. In the wall of the described aneurysm, there were seen single calcium inclusions. The aneurysm had a gap connection, up to 6 mm in size, with superior mesenteric vein (SMV), which was dilated up to 49−50 mm, and deformed; it actively contrasted in an arterial phase due to a marked shunt from SMA. The portal vein diameter was 20 mm. No evidence on portal thrombosis was found. The spleen size was 120×45 mm. The splenic vein diameter was 7 mm. There was defined a marked network of pathologically dilated mesenteric veins and a large number of protuberant varicosities in the duodenal and jejuna walls, as well as in the pancreatic head.*

*Taking into consideration the patient’s critical condition and a threat of recurrent major bleedings from esophageal and gastric veins, the patient required urgent curative measures. However, due to the severity, and the presence of portal hypertension with mesenteric varicosity of the small intestine and the stomach, and considering the previously performed medial laparotomy and the pseudo-aneurysm localization in the mesentery resulted from an open surgery, it was decided to abstain and perform an X-ray endovascular procedure.*

**Figure 2:**
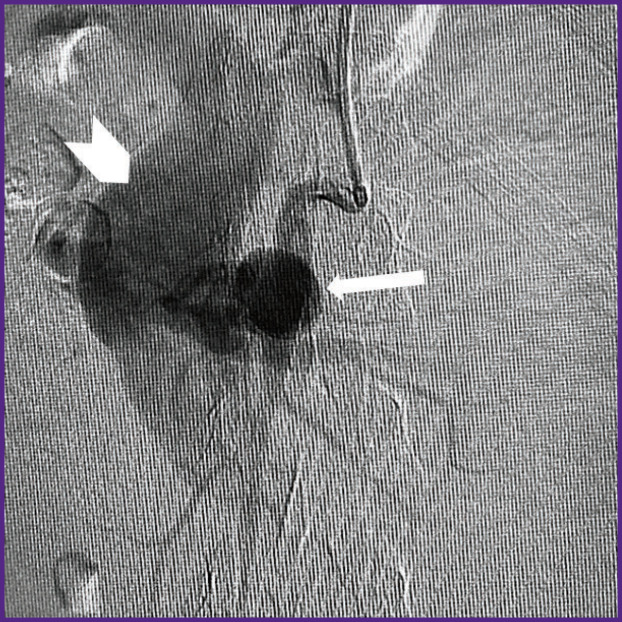
Patient S., selective arteriogram of the superior mesenteric artery Contrast medium is filling a pseudo-aneurysm (*narrow arrow*), and through the aneurysm — into the arteriovenous fistula of a dramatically dilated superior mesenteric vein (*wide arrow*)

*In OR, the patient was given local anesthesia, and SMA was selectively catheterized by a puncture through the left brachial artery, and arteriography was performed. The latter confirmed CT-angiography findings of SMA aneurysm, 32×35 mm in size, as well as the presence of AVF with marked shunt from the arterial bed to SMV (Figure 2). The SMA trunk was moderately dilated: from the artery mouth to the saccular pseudo-aneurysm its diameter being 8 mm, below the aneurysm the diameter was 6–4 mm. It also confirmed the significant dilation of SMV — up to 48–50 mm along the entire length from AVF localization to SMV and the splenic vein confluence.*

**Figure 3:**
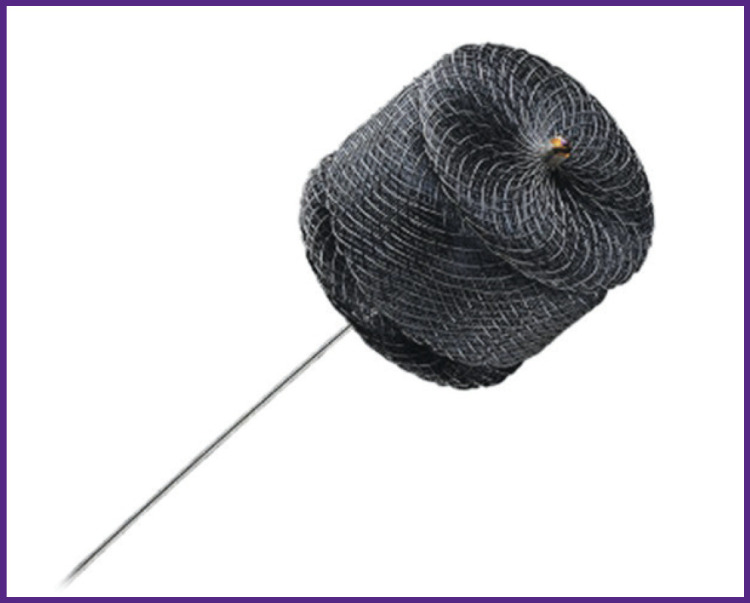
Amplatzer Vascular Plug II occluder (USA) to embolize peripheral vessels It is made of two-layer nitinol mesh, and includes three segments

*The aneurysm ostium inside SMA was engaged with a guiding catheter RDC F6 (Cordis, USA), and we made an attempt to embolize the aneurismal cavity by a coil, 10×8 mm (COOK Medical, USA). The coil inserted in the aneurismal cavity, due to a high-speed blood flow between the mesenteric artery and the vein, migrated from the aneurysm cavity through the arteriovenous connection into SMV and further to one of the portal vein branches. We had to abandon the embolization technique. It was decided to close AVF by analogy with the technique used to eliminate the cardiac septum defect using a braided nitinol occluder. An endovascular closing device — Amplatzer Vascular Plug II (AGA Medical Corporation, USA), 14×10 mm in size, was implanted (Figure 3).*

**Figure 4:**
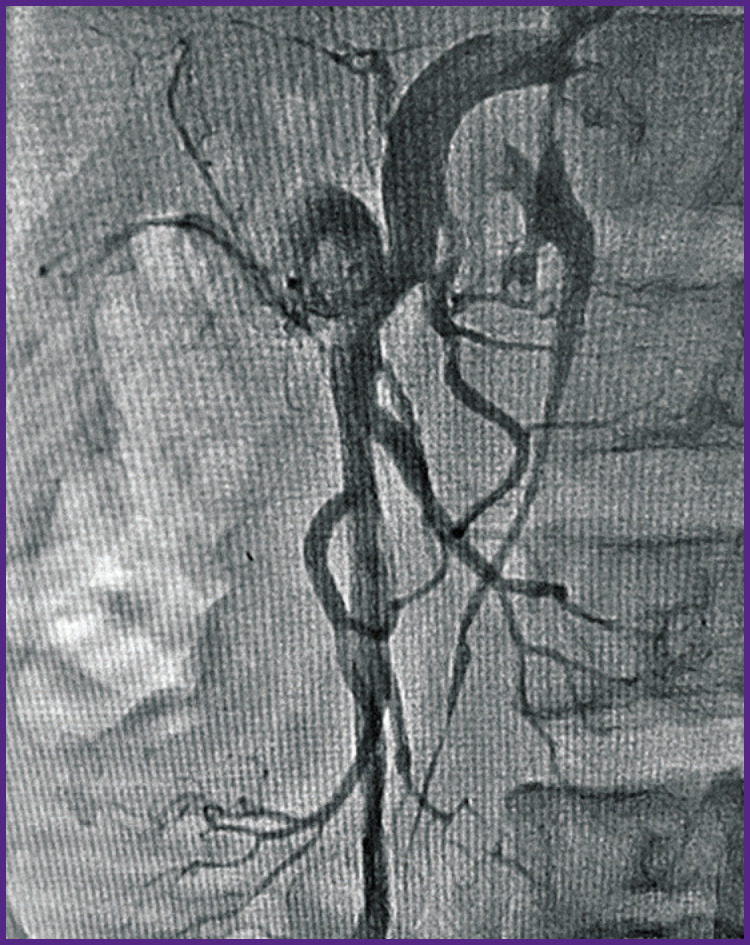
Patient S., arteriogram immediately after the occlusion of arteriovenous fistula and pseudo-aneurysm Shunting from the superior mesenteric artery into the superior mesenteric vein is ceased

*For the first time ever in the established endovascular practice the occluder was implanted in the aneurismal cavity of the mesenteric artery and mesenteric AVF. A distal disc of the occluder was expanded in the SMV lumen. The medium and proximal occluder parts were expanded in the pseudo-aneurysm cavity. Shunting from SMA into SMV discontinued immediately after the occluder was implanted (Figure 4). An endovascular intervention was completed by withdrawing the catheters from bloodstream, hemostasis, and bandaging the puncture area of the brachial artery (aseptic dressing).*

*In the immediate postoperative period Blackmore tube was stepwise blown out and withdrawn. On day 10 after the surgery, a comparison EGDS was performed, it revealing tortuous trunks of varicose veins, up to 4 mm in diameter, partially collapsed in air insufflation, the mucosa above them being unchanged. No recurrent bleeding signs were found.*

**Figure 5:**
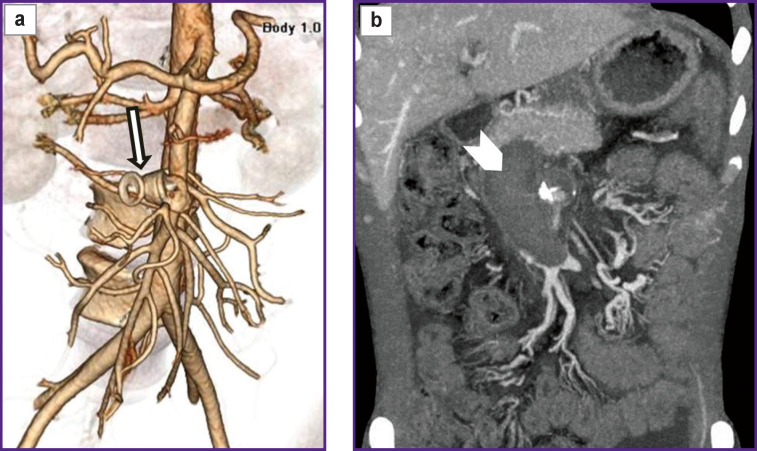
Patient S., MSCT-angiograms on day 10 after endovascular occlusion of the mesenteric arteriovenous fistula: (а) 3D reconstruction — the shunting from the superior mesenteric artery into the superior mesenteric vein is ceased; in the pseudo-aneurysm cavity there can be found the occluder body (*narrow arrow*); (b) a portal phase, coronary view — in the pseudo-aneurysm projection there is a bright shadow of metal density (occluder); the superior mesenteric vein trunk is occluded by a thrombus (*wide arrow*)

*A comparison abdominal MSCT was performed (Figure 5). It showed persisting ascites and portal hypertension symptoms. In the portal vein trunk lumen switching to SMV there was revealed an extended contrast defect — a clot obstructing the entire vein lumen. Intestinal branches of SMV were patent, draining through a network of feebly marked portal-systemic anastomoses. Moreover, thrombosis extended to intrahepatic branches of the portal vein, where clots occupied more than half of the lumen.*

*In the occlusion area of a pathologic arteriovenous anastomosis there could be seen the artifacts of metal density from the occluder. SMA patency was unimpaired. The pseudo-aneurysm cavity was thrombosed, except for the SMA wall defect area, where a cavity, 13×9 mm in size persisting, it indicating incomplete thrombosis of SMA pseudo-aneurysm. There could be visualized a foreign body of the right liver lobe (framing coil), gallstones.*

*Because of a starting thrombosis of the superior mesenteric vein and the portal vein, there was provided a complex anticoagulant therapy with a good clinical effect; a dynamic study showed partial recanalization of the veins. No recurrent gastrointestinal bleedings occurred. The patient was discharged from the inpatient department in a satisfactory condition 22 days after the endovascular intervention.*

**Figure 6:**
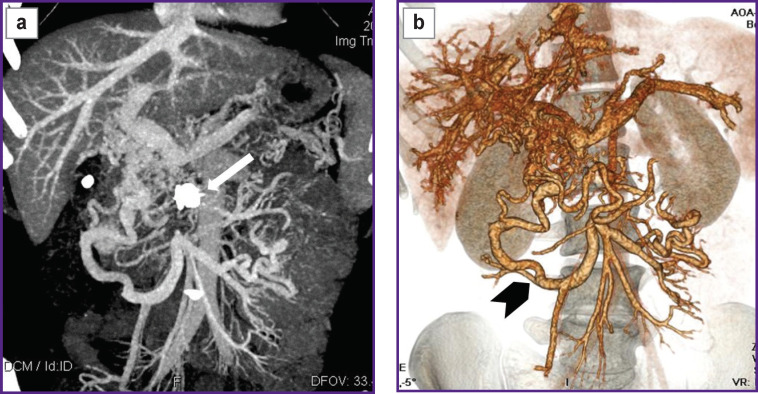
Patient S., abdominal MSCT 95 days after mesenteric arteriovenous fistula occlusion: (а) MIP, coronary view; the occluder body is located in the pseudo-aneurysm projection (*narrow white arrow*); the superior mesenteric vein does not contrast; there is visualized a network of collateral mesenteric veins with no indirect signs of portal hypertension; (b) 3D reconstruction — a system of collaterals of mesenteric veins (*wide black arrow*); the superior mesenteric vein is occluded

*The patient was followed up 4 months later. EGDS findings had no evidence for esophageal varices. A comparison MSCT was performed (Figure 6). The pseudo-aneurysm of SMA was thrombosed, except for a small area in the aneurysm orifice. There were no signs of blood shunting between SMA and SMV. No free fluid was found in the abdominal cavity and the true pelvis. The liver was not enlarged, its contours being clear and smooth. A foreign body (framing coil) of high density, 17×9 mm in size, was visualized in the right liver lobe (S6). At other levels the parenchyma structure was homogeneous. Intrahepatic bile ducts were not dilated. The portal vein trunk, its intrahepatic branches and the splenic vein were of ordinary diameters, no thrombosis signs were revealed. SMV below the confluence, throughout of 33 mm, was occluded by a thrombus. Below the occlusion, the intestinal branches were patent, slightly dilated, drained through a network of well-marked porto-portal anastomoses. There were no portal hypertension signs found. Esophageal and gastric veins in front of AVF occlusion were not contrasted.*

*In the occlusion area of the pathological arteriovenous fistula there were visualized the artifacts from the occluder. SMA patency was not impaired. The pseudo-aneurysm cavity was thrombosed, except for a small neck area with the contrasted cavity, 10×10 mm in size. The spleen was 117×45 mm in size, its contours being clear, smooth. The splenic vein was 8 mm in diameter. The abdominal aorta and the inferior vena cava had no abnormalities.*

*Outpatient observation is to be continued.*

## Results and Discussion

An arteriovenous fistula is a pathological congenital or artificially created communication between an artery and a vein, through which blood flows by passing a capillary network. By localization, about 47% of such fistulas are in the upper and lower limbs, 43% — in the head and chest areas. In 10% cases AVFs have other localization including the abdominal cavity, as well as mesenteric AVF. Mesenteric AVFs as surgical complications or resulting from wounds occur quite rarely. Currently, about 200 AVF between the abdominal vessels have been described so far [2].

Arteriovenous fistula with anastomoses of large diameters result in marked shunting from arterial vessels with systemic pressure into venous bed, where initial pressure does not exceed 3–7 Hg mm. As a result, arteriovenous fistula eventually dilates to the extent of an aneurismal size, and a marked volume overload of the venous bed. In case AVF is located between systemic arteries and veins, there develops the volume overload of the right cardiac cavity and lesser circulation vessels that in the end forms secondary pulmonary hypertension. The described clinic picture is characteristic of AVF occurring between the systemic arteries and veins, between the aorta and the portal vein, or its major feeders [4, 5].

If AVF is located between mesenteric arteries and veins, hemodynamic compromises develop in other way. Arteriovenous blood shunt into the portal vein forming in traumatic anastomosis resulted from a surgery on the intestine or an abdominal wound, is accompanied by hemodynamic arterialization in the trunk and branches of the portal vein, and increased pressure in the vein up to the systemic level [1, 6, 7]. Furthermore, a clinical presentation changes dramatically. When describing patients with posttraumatic AVF of mesenteric arteries and veins and hypertension in the portal vein system, Radonić et al. [8] specified such symptoms as low fever, anterior abdomen wall thrilling and murmur on auscultation in the epigastric area. The authors mentioned frequent abdominal pains especially after meals, flatus, loose stool, nausea, heartburn, vomiting, convulsions, oliguria. The mentioned pathological changes of portal hemodynamics and esophageal and gastric varicosity can result in patient’s death of upper gastrointestinal bleeding, even ten or more years after AVF formation [9].

The data reported in the represented articles for all intents and purposes were proved by our patient’s case history. Within a 54-month postoperative period after the wound and an urgent surgery — the resection of impaired small intestinal area — that saved the patient’s life, there developed a giant pseudo-aneurysm and AVF between SMA and the vein with a marked shunt from the arterial bed into the portal vein system, and portal blood flow arterialization. For a great while it manifested itself clinically in no way. However, after formed bypaths of portal-systemic venous outflow and esophageal and gastric varicosity, there were several upper gastrointestinal bleeding events. It also completely corresponds to the data reported in the mentioned articles.

Currently, apart from clinical signs of the volume overload of the portal system and liver insufficiency under portal hypertension, radiodiagnostics plays a key role in mesenteric AVF diagnosis. Most authors suggest duplex scanning and MSCT with contrast enhancement (MSCT-angiography) as screening methods [4, 8, 10]. An invasive survey and selective angiography are used to keep data current or as a treatment stage [4, 5, 11]. MSCT-angiography provides comprehensive anatomical data on the size of afferent and deferent vessels, AVF size, and enables to determine an approach to a pathological anastomosis, and find an optimal surgical technique. In our case it was used to choose an intravascular approach and select an occluder type and size.

Due to the variability of the anatomy and size of such intervascular fistulas, possible aneurysm formation, portal hypertension and its complications, any patient therapy requires individual, sometimes unique, solutions, a nonstandard surgical approach to AVF separation. Classical therapy for iatrogenic and traumatic AVF between visceral vessels is surgical separation of fistula with the excision of aneurysmatically dilated arterial and venous collectors. However, an open surgery, which is performed as a secondary intervention, a long period of time after the first surgery, is technically difficult due to adhesions in the abdominal cavity and presents a risk of damaging major vessels [3, 4, 8].

In recent decades, there has been suggested a variety of less traumatic procedures to isolate arteriovenous anastomoses. The list of such surgeries is as follows: a fistula isolation by implanting a drug-eluting stent (graft) in the damaged artery lumen [5, 12, 13]; fistula occlusion by framing coils [14–17], adhesive compositions, by Onyx [18]; the occlusion of afferent arteries by nitinol braided occluders — an amplatzer septal occluder, an amplatzer vascular plug, and others [2, 11, 17, 19–23]. In these cases invasive angiography is used after diagnostic procedures as a navigation technique and to control the effect of an endovascular intervention.

Determining a surgery technique, an approach to an arteriovenous anastomosis area, and the separation method selection to separate the arterial and venous beds depend on AVF anatomical location, an afferent artery size, the presence of aneurysms in the fistula area, the blood flow velocity and volume through the anastomosis.

An arteriovenous fistula between superior mesenteric artery and vein, our patient had for a long time, was complicated by giant pseudo-aneurysm formation. Its location deep in mesenteric tissues, apparently surrounded by adhesions after the first surgery, combined with portal hypertension and a developed network of porto-caval venous collaterals almost eliminated the possibility of an open surgery.

A decision on transcatheter AVF occlusion in an ‘inconvenient’ for endovascular procedures SMA lumen (6–8 mm in diameter) was due to the difficulties related to inserting carrying catheters and occluders in the artery lumen to the aneurysm orifice. Therefore, at first it was decided to use framing coils, which were supposed to fill the pseudo-aneurysm cavity and that way cease arteriovenous shunt into SMV.

However, an attempt of using coil occluders in our observation failed due to a high-speed blood flow through an arteriovenous fistula. The first coil implanted in the aneurysm migrated into the portal vein branch of the liver S6. The efforts to remove the coil appeared to be vain. Then it was decided to close AVF between the superior mesenteric artery and vein using an occluder: Amplatzer Vascular Plug II (USA), it was for the first time in endovascular practice when an occluder was implanted in this location (by analogy with the occlusion of cardiac septum defect), and using the occluder design characteristics.

No descriptions of similar occlusion techniques for mesenteric AVF complicated by a developed pseudo-aneurysm of the afferent artery and aneurismal dilation of the defferent vein were found. The distal disc was opened and put into SMV lumen. Other occluder elements expanded in the pseudo-aneurysm lumen in turns occupying nearly all the volume, while the fistula itself appeared to be ‘locked’ between the occlude discs.

A unique experience of using an occluder, some minutes after its implantation, enabled to stop arteriovenous shunting and break the pathological mechanism of forming and maintaining high blood pressure in the portal vein system. The arteriovenous shunt termination, in its turn, ceased further formation of esophageal and gastric varicosity, and their bleedings. A stable therapeutic effect was achieved.

## Conclusion

For the first time in practice, transcatheter implantation was performed to occlude mesenteric arteriovenous fistula: a braided nitinol occluder Amplatzer Vascular Plug II (USA) was implanted according to the occlusion techniques used for septal defects. It enabled to demonstrate the capabilities of an endovascular method to treat mesenteric arteriovenous fistulas complicated by a giant pseudo-aneurysm and portal hypertension.
